# Attenuated Organomagnesium Activation of White Phosphorus**

**DOI:** 10.1002/anie.201503065

**Published:** 2015-05-26

**Authors:** Merle Arrowsmith, Michael S Hill, Andrew L Johnson, Gabriele Kociok-Köhn, Mary F Mahon

**Affiliations:** Department of Chemistry, University of Bath Claverton Down, Bath, BA2 7AY (UK) E-mail: msh27@bath.ac.uk

**Keywords:** cluster compounds, magnesium, P_4_ activation, phosphorus, structure elucidation

## Abstract

Sequential reactions between a 2,6-diisopropylphenyl-substituted β-diketiminato magnesium *n*-butyl derivative and P_4_ allow the highly discriminating synthesis of unusual [*n*Bu_2_P_4_]^2−^ and [*n*Bu_2_P_8_]^2−^ cluster dianions.

White phosphorus is the starting point in the synthesis of many organophosphanes and phosphorus-containing molecules. As a consequence, the controlled activation of P_4_ has attracted considerable attention.[1] While notable metal-free examples of P_4_ activation are provided by Bertrand’s use of nucleophilic N-heterocyclic carbenes for sequential P–P activation of the tetrahedral molecule,[2] the majority of approaches have utilized either reactive transition-metal or main-group reagents. Typical transition-metal-based activation can induce the cleavage of bonds within P_4_ and also the aggregation of smaller fragments to form P_*n*_ (*n*>4) cluster anions. Cummins, for example, has shown that disproportionation of a niobium(IV) tris(enolate) in the presence of P_4_ provides the {P_8_} cluster compound **1**,[3] while in a very recent report, Bergman and Arnold have described a trinuclear β-diketiminato μ-P_12_ niobium complex, which was isolated as one of multiple minor by-products during the reaction of the Nb^V^ complex (^Dipp^BDI)Nb(N*t*Bu)Me_2_ (^Dipp^BDI=HC{C(Me)_2_N(2,6-*i*Pr_2_C_6_H_3_)}_2_) with H_2_ in the presence of P_4_.[4] Notably, this latter reaction gave a compound containing a cyclo-P_4_ dianion as its predominant reaction product and, in some respects, the irrationality of the formation of both of these compounds is typical of the synthesis of high-nuclearity {P_*n*_} clusters.[1f]


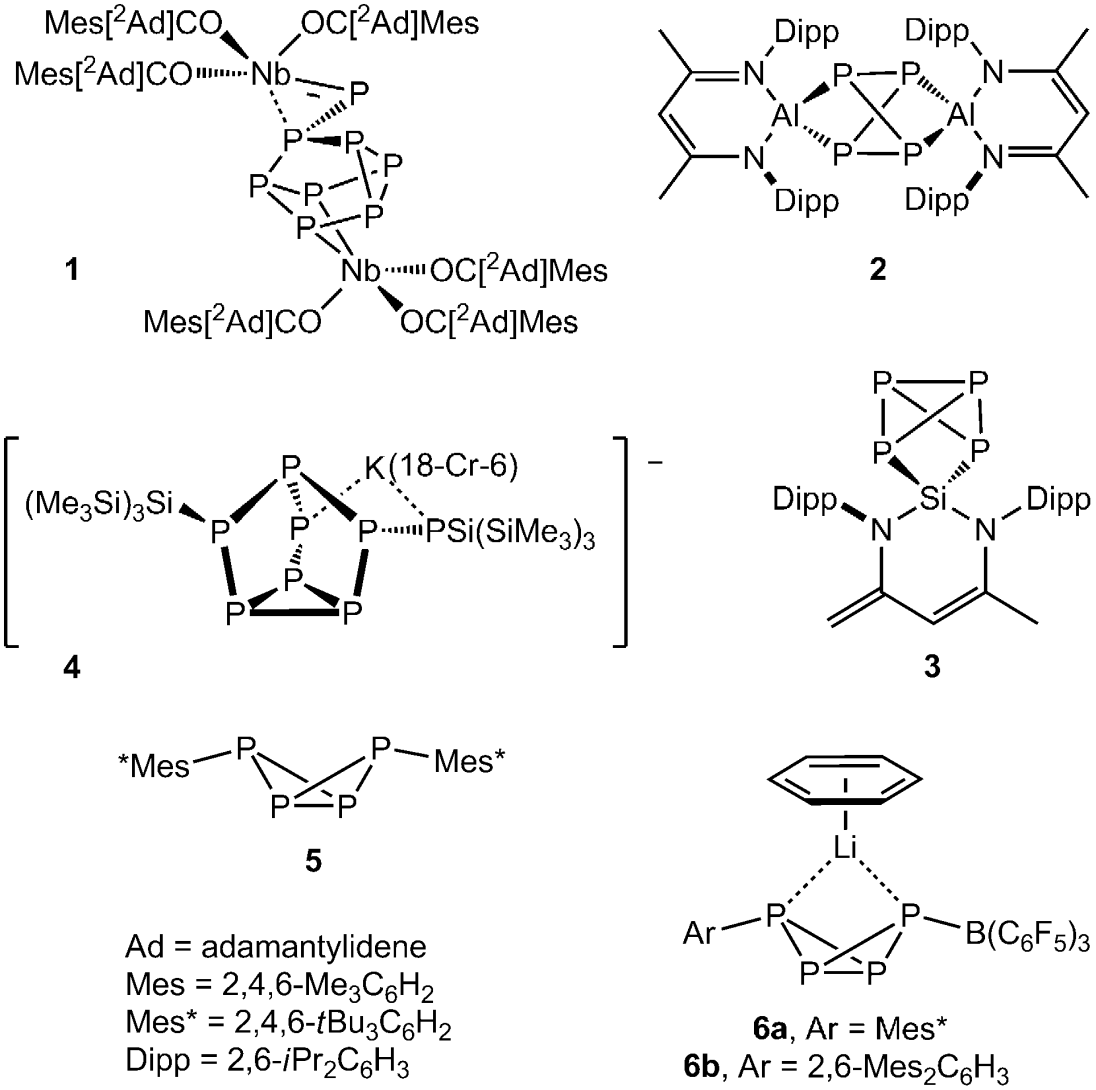


Reductive chemistry to *cyclo*-P_4_ anions has also been described through the formal oxidative addition of P_4_ to low-oxidation-state Group 13 and 14 derivatives (for example, compounds **2** and **3**),[1c, 5, 6] and there is precedent for the activation of P_4_ through its interaction with transition-metal hydrides and alkyls.[7] Well-defined reactions with anionic Group 14 derivatives of elements from Groups 1 and 2 are, however, uncommon and dominated by the use of highly sterically demanding carbon or silicon reagents. Wright and co-workers described the synthesis of the [R_2_P_8_]^2−^ cluster dianion (**4**) derived from a {P_7_}^3−^ Zintl ion core through the reaction of the very bulky hypersilyl potassium reagent with P_4_.[8] The disubstituted butterfly compound **5** (Mes*=2,4,6-*t*Bu_3_C_6_H_2_) and the diphosphene Mes*P=PMes* were similarly isolated from the reaction of Mes*Li and P_4_ in the presence of Mes*Br.[9] Production of compound **5** presumably occurs through initial addition of Mes*Li across a P–P bond within the P_4_ unit and the intermediacy of the consequent organopolyphosphorus anion. The existence of species of this latter type has recently been confirmed by Lammertsma and co-workers, who reported that the B(C_6_F_5_)_3_-stabilized lithium bicyclo[1.1.0]tetraphosphabutane species **6 a** and **6 b** could be isolated from reactions of P_4_ and sterically demanding lithium aryls in the presence of the borane Lewis acid.[10] Although similar but indiscriminate reactivity was posited some 50 years ago during reactions with simple Grignard reagents,[11] the activation of white phosphorus by nucleophilic Group 2 reagents is even less widespread than that by the alkali metals. Our own research interests lie in the development of a defined bond activation and homogeneous catalytic chemistry for the earth-abundant and environmentally benign alkaline earth elements.[12] As a first step toward the functionalization of white phosphorus catalyzed by a Group 2 metal, we present in this contribution the results of our initial study, which indicate that remarkable kinetic control during P_4_ activation is achievable with organomagnesium reagents (Scheme 1). Key to the success of this chemistry is the application of the sterically demanding ^Dipp^BDI supporting ligand, which was employed in the syntheses of Arnold and Bergman’s aforementioned niobium systems,[4] compounds **2** and **3**, and very recently described binuclear cobalt(I) and copper(I) species containing a P_4_-derived tetraphospacyclobutadiene and an unactivated P_4_ ligand respectively.[13]

**Scheme 1:**
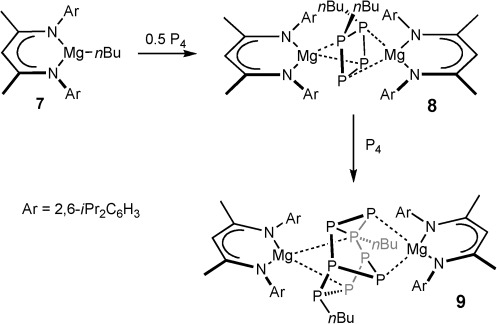
Synthesis of the complexes described herein.

An initial reaction between an equimolar quantity of P_4_ and the β-diketiminato *n*-butylmagnesium complex, [(^Dipp^BDI)Mg*n*Bu] (**7**), resulted in the smooth consumption of the magnesium starting material to provide a single new species (**8**) characterized by two mutually coupled doublet resonances at *δ* −25.6 and −195.5 ppm (^1^*J*_P-P_=99.6 Hz) in the resultant ^31^P{^1^H} NMR spectrum. Notably, although compound **8** formed with only 50 % consumption of P_4_, some evidence of further reaction was apparent on continued monitoring (see below). Repetition of this reaction with the reagent quantities adjusted to reflect the apparent 2:1 reaction stoichiometry resulted in the complete consumption of both P_4_ and **7** and the sole production of compound **8**, which also comprised a single set of temperature-invariant (from −90 to +90 °C) *n*-butyl and (^Dipp^BDI) ligand environments in the corresponding ^1^H and ^13^C NMR spectra. The origin of these observations was resolved through the isolation of colorless single crystals of compound **8** suitable for X-ray diffraction analysis from an *n-*hexane solution at low temperature, the results of which are displayed in Figure 1. The molecular structure of **8** comprises a dinuclear magnesium complex of an unprecedented nonplanar [*n*Bu_2_P_4_]^2−^ dianion, which may be considered as arising from the formal addition of two magnesium *n*-butyl fragments to, and the consequent activation of two P-P single bonds within, the P_4_ tetrahedron. The P–C bonds to the *n*-butyl chains are located on the adjacent P1 and P2 atoms, while the magnesium centers each coordinate to one of these alkylated phosphorus atoms and the phosphorus atom located at the opposite diagonal corners of the tetraphosphorus unit. The adoption of a square array is prevented by a distinct pyramidalization at each phosphorus center such that the P-P-P angles are compressed to about 80°. The P3–P4 bond length [2.2647(7) Å] is significantly elongated in comparison to the interatomic separation between the alkylated phosphorus atoms [P1–P2 2.1762(7) Å] but is comparable to the P–P distances observed in molecules containing the [P_4_]^4−^ anion, such as compound **2** [mean 2.29 Å].[5] The Mg–P distances to P3 and P4 [Mg1–P3 2.5625(7), Mg2–P4 2.5592(7) Å] are also shorter than those to P1 and P2 [Mg1–P1 2.6336(7), Mg2–P2 2.6652(7) Å] and we interpret both of these structural features to indicate that the distribution of negative charge is polarized toward the non-alkylated P3 and P4 centers and that the [*n*Bu_2_P_4_]^2−^ unit as a whole may be considered as derived from the formal addition of two *n*-butyl carbenium ions to the [P_4_]^4−^ constituent anion of compound **2**.

**Figure 1:**
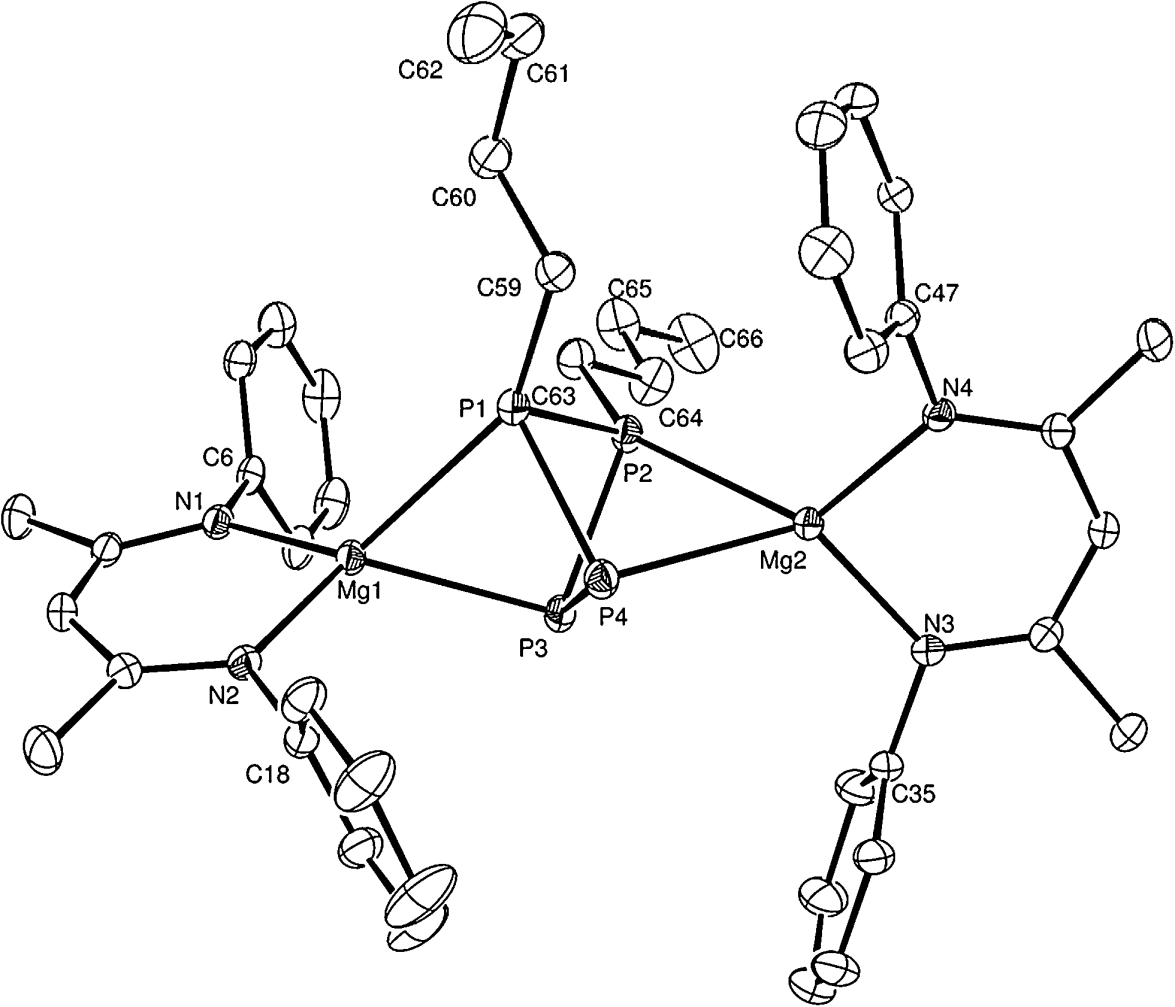
ORTEP representation (30 % probability ellipsoids) of compound 8.[17] Hydrogen atoms and isopropyl groups were removed for clarity. Selected bond lengths [Å] and angles [°]: Mg1–N1 2.0462(15), Mg1–N2 2.0465(15), Mg1–P3 2.5625(7), Mg1–P1 2.6336(7), Mg1–P4 3.1607(7), Mg2–N4 2.0424(15), Mg2–N3 2.0466(15), Mg2–P4 2.5592(7), Mg2–P2 2.6652(7), P1–C59 1.8584(19), P1–P2 2.1762(7), P1–P4 2.2127(7), P2–C63 1.8575(19), P2–P3 2.2108(7), P3–P4 2.2647(7); N1-Mg1-P3 126.57(5), N2-Mg1-P3 122.72(5), N1-Mg1-P1 127.76(5), N2-Mg1-P1 122.16(5), P3-Mg1-P1 67.14(2), N1-Mg1-P4 167.48(5), N2-Mg1-P4 99.06(5), P3-Mg1-P4 45.131(17), P1-Mg1-P4 43.731(16), N4-Mg2-N3 93.52(6), N4-Mg2-P4 126.28(5) N3-Mg2-P4 121.83(5), N4-Mg2-P2 121.40(5), N3-Mg2-P2 130.79(5), P4-Mg2-P2 66.130(19).

The previously noted onward reaction of compound **8** with additional P_4_ (see above) prompted us to carry out a further reaction of an equimolar quantity of P_4_ and compound **8**. The reaction in benzene at room temperature provided the slow and simultaneous consumption of the starting materials and direct conversion to a single new compound (**9**), which is characterized by the emergence of eight mutually coupled signals in the ^31^P NMR spectrum. The corresponding ^1^H and ^13^C NMR spectra indicated that this new compound comprised two differentiated groups of resonances indicative of two separate *n*-butyl and (^Dipp^BDI) ligand environments. Compound **9** could also be synthesized directly through the slow (7 days) reaction of compound **7** with an equimolar amount of P_4_ at room temperature or by heating of a similar reaction at 60 °C for 2 days. In this latter case, compound **9** crystallized as colorless single crystals suitable for a further X-ray diffraction analysis, allowing the resolution of the solution NMR spectroscopic observations. The result of this analysis is shown in Figure 2 and highlights that the disposition of the phosphorus and magnesium centers within compound **9** is consistent with the coupling pattern observed in the solution-state ^31^P NMR spectrum and the multiplicity of signals arising in the ^1^H and ^13^C NMR data. The structure comprises two (^Dipp^BDI) magnesium units bonded to a [*n*Bu_2_P_8_]^2−^ cluster dianion derived from the formal reductive coupling of two neutral P_4_ units. Although the rationality of this {P_8_} cluster synthesis from the combination of two {P_4_} fragments appears to be unique, its structure may be considered as a heptaphosphanorbornane fused to an *exo*-oriented cyclotriphosphane ring and a constitutional isomer of the {P_8_} cage within the niobium(V) species [Ph_2_CP_8_Nb(OC[^2^Ad]Mes)_3_], which was derived through treatment of compound **1** with benzophenone.[14] The single tris(enolato) niobium dication and the {Ph_2_C} unit of this previously described species is replaced in the present case by two (^Dipp^BDI) magnesium monocations and two *n*-butyl substituents. The magnesium centers contact the {P_8_} cage structure in *endo* [Mg1] and *exo* [Mg2] configurations with respect to the P4 bridgehead and provide Mg–P distances of 2.575(3) Å [P4–Mg2], 2.625(3) Å [P8–Mg2], 2.594(3) Å [P2–Mg1] and 2.831(3) Å [P7–Mg1]. Although these latter values indicate some variability in the charge donation across the cage structure, it should be noted that the Mg–P contacts are all within the range expected [2.45 to 2.99 Å] from a search of the Cambridge structural database.[15] All of the P–P distances within this new cluster are also consistent with P–P single bonds and range between 2.096(3) Å [P3–P4] and 2.262(4) Å [P5–P6].

**Figure 2:**
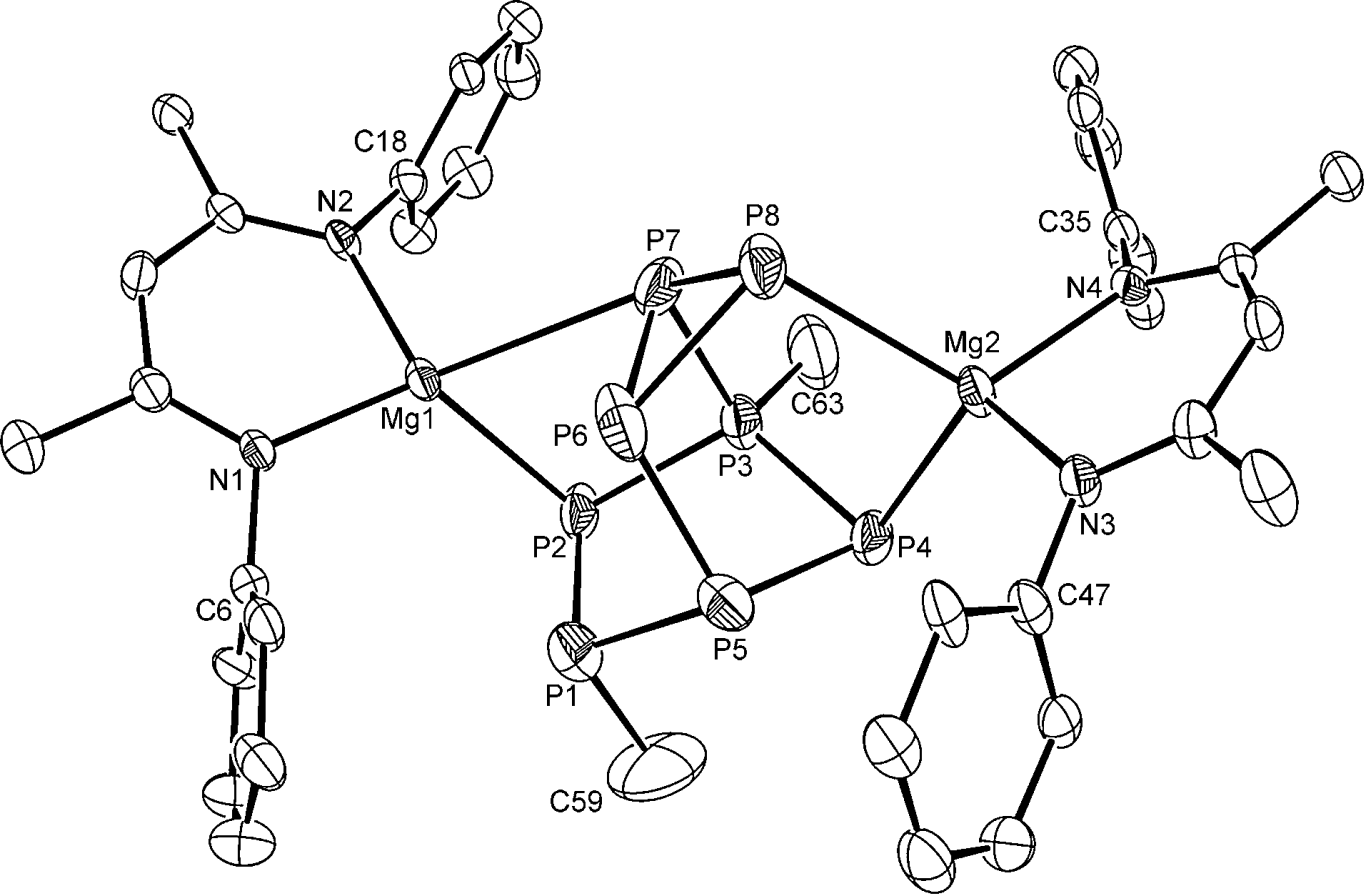
ORTEP representation (30 % probability ellipsoids) of compound 9.[17] Hydrogen atoms, isopropyl methyl groups and *n*-butyl groups aside from the P-bonded C59 and C63 atoms were removed for clarity. Selected bond lengths [Å] and angles [°]: P1–P5 2.195(3), P2–P3 2.160(2), P2–Mg1 2.594(3), P3–P4 2.096(3), P3–P7 2.186(3), P4–P5 2.222(3), P4–Mg2 2.575(3), P5–P6 2.262(4), P6–P7 2.170(4), P6–P8 2.185(3), P7–P8 2.153(3), P7–Mg1 2.831(3), P8–Mg2 2.625(3) Mg1–N2 2.046(5); P2-P1-P5 105.23(12), P1-P2-P3 96.79(11), P1-P2-Mg1 90.77(11) P3-P2-Mg1 97.47(9), P4-P3-P7 113.74(12), P2-P3-P7 97.95(10), P3-P4-P5 88.12(10), P3-P4-Mg2 101.38(10), P5-P4-Mg2 91.12(11), P1-P5-P4 102.66(14), P1-P5-P6 92.00(12), P4-P5-P6, 105.80(12), P7-P6-P8 59.27(11), P7-P6-P5 106.69(12).

Subsequent preliminary investigations indicated that the kinetic control exerted during the syntheses of compounds **8** and **9** is an apparent consequence of the steric demands of both the supporting (^Dipp^BDI) platform and the reactive *n*-butyl co-ligand. Analysis by ^31^P NMR spectroscopy of a reaction between [(^Dipp^BDI)MgH]_2_ and half a molar equivalent of P_4_ provided a mixture of several phosphorus-containing species. Although a minor component comprised broadened resonances at *δ* −3.5 and −159 ppm reminiscent of those observed for compound **8**, the major product of this reaction was characterized by a triplet signal observed at *δ* −284.8 ppm (^1^*J*_P-H_=45 Hz). This latter species was identified as the trimeric primary magnesium phosphide, compound **10**, through a further X-ray analysis performed on single crystals produced by fractional crystallization of the reaction mixture (Figure 3 a). Analytically pure samples of compound **10**, notable as the first primary phosphide of a Group 2 element to have been structurally characterized, were found to be unstable once redissolved in [D_8_]toluene. Monitoring by ^1^H and ^31^P NMR spectroscopy at room temperature evidenced the onset of a redistributive process to as yet unidentified magnesium-containing species and the production of PH_3_, which was observed as a binomial quartet at *δ* −245.0 ppm in the ^31^P NMR spectrum.

**Figure 3:**
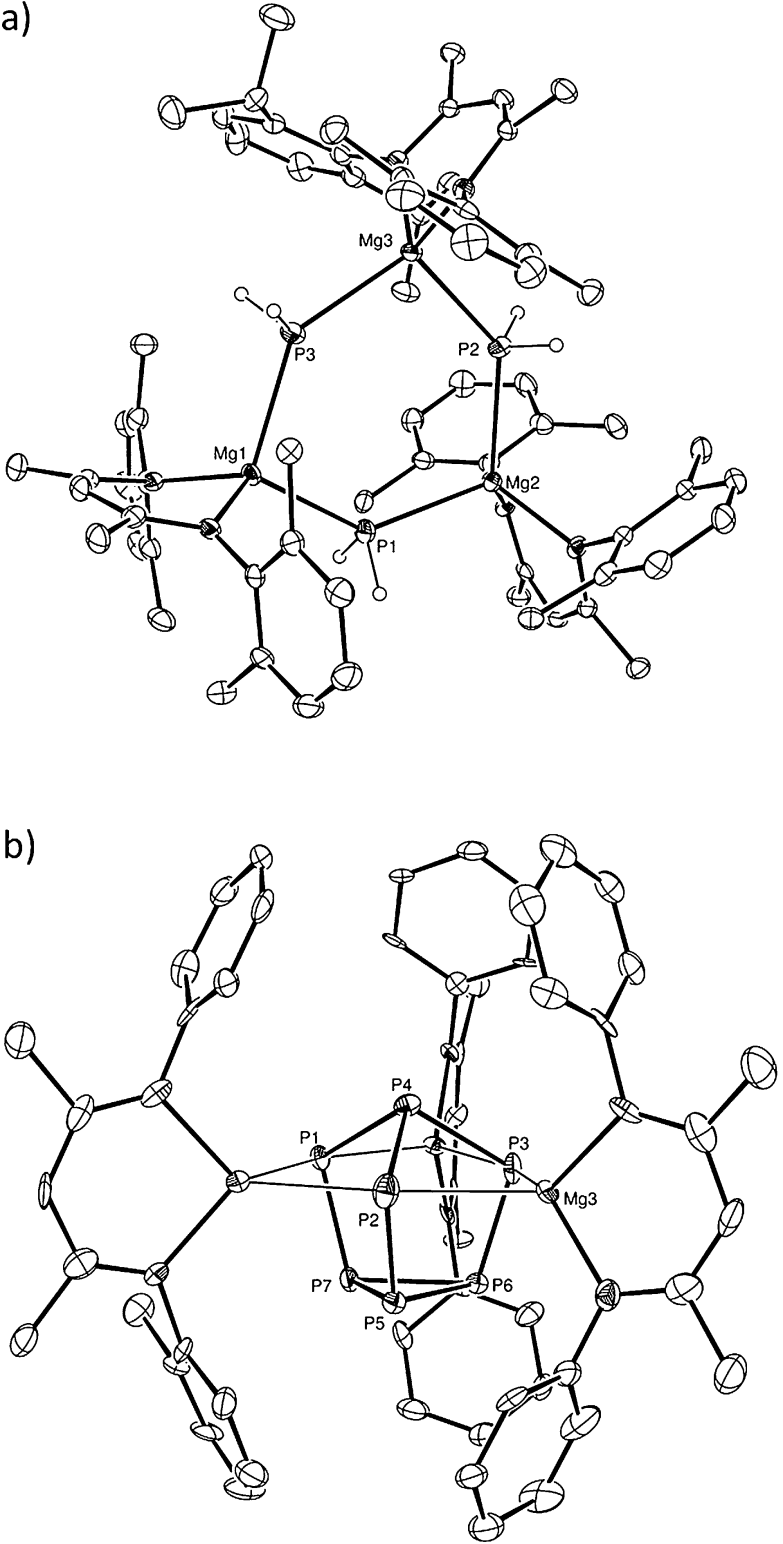
ORTEP representations (30 % probability ellipsoids) of a) compound 10 with hydrogen atoms (except those attached to P1, P2, and P3) and isopropyl methyl groups removed for clarity, and b) compound 11 with hydrogen atoms and mesityl methyl groups removed for clarity.[17]

In a similar manner, monitoring by NMR spectroscopy of a reaction analogous to that employed in the synthesis of compound **8**, but with replacement of the *N*-{2,6-*i*Pr_2_C_6_H_3_} groups of the (^Dipp^BDI) ligand of **7** by less sterically demanding *N*-{2,4,6-Me_3_C_6_H_2_} substituents (^Mes^BDI), indicated less discriminating behavior and reactivity, which again resulted in the formation of multiple products. A further reaction performed between [(^Mes^BDI)Mg*n*Bu] and P_4_ in a 2:3 molar ratio also provided a complex mixture containing at least three new phosphorus-containing compounds. Fractional crystallization from this solution and a resultant single crystal X-ray diffraction analysis of compound **11** (Figure 3 b) provided some insight into the nature of these processes. Compound **11** may be viewed as a [P_7_]^3−^ Zintl ion cage reminiscent of compound **4** but decorated with three β-diketiminato magnesium units. We suggest, therefore, that the formation of this phosphorus cage constitutes a thermodynamic sink during the solution comproportionation of magnesium polyphosphorus species of varying nuclearity.[16]

In conclusion we have shown that the controlled, sequential organomagnesium-mediated activation of white phosphorus may be achieved through selection of an appropriate combination of supporting and reactive co-ligands. We are continuing to explore the parameters of this reactivity and will report our findings in due course.

## Supporting Information

As a service to our authors and readers, this journal provides supporting information supplied by the authors. Such materials are peer reviewed and may be re-organized for online delivery, but are not copy-edited or typeset. Technical support issues arising from supporting information (other than missing files) should be addressed to the authors.

miscellaneous_information
