# Supplementary material for: Attenuated Organomagnesium Activation of White Phosphorus
Source: Angew Chem Int Ed Engl. 2015 May 26;54(27):7882–5. doi: 10.1002/anie.201503065 (PMC4648029; doi:10.1002/anie.201503065)
Supplement: Supplementary file 1 — miscellaneous_information [file anie0054-7882-sd1.pdf]

Angewandte Chemie
Eine Zeitschrift der Gesellschaft Deutscher Chemiker

Supporting Information

Attenuated Organomagnesium Activation of White Phosphorus**

Merle Arrowsmith, Michael S. Hill, Andrew L. Johnson, Gabriele Kociok-Köhn, and Mary F. Mahon**

anie_201503065_sm_miscellaneous_information.pdf

Experimental Notes

General Procedures and Materials

All manipulations were carried out under an inert atmosphere of argon utilizing standard glovebox and Schlenk line techniques. J. Youngs tap NMR tubes sealed in a glovebox were employed for NMR spectroscopy which was performed on a Bruker AV-300 spectrometer or a Bruker AV-400 spectrometer with spectra referenced to residual solvent peaks. Spectra were recorded at 298 K unless otherwise noted. X-ray diffraction data for compounds **8** and **10** were collected on a Nonius Kappa CCD with a low temperature device at 150 K, utilizing Mo-Kα radiation monochromated with graphite (λ = 0.71070 Å), while data for compounds **9** and **11** were recorded on an Agilent Xcalibur. Data were processed with the Nonius software,[i, ii] with structure solution and refinement using XSeed, SHELXS and SHELXL[iii] and visualised utilising Ortep 3.[iv] Solvents were dried with an Innovative Technologies PureSolv MD SPS (n-hexane, toluene) or by distillation from potassium/benzophenone (THF) and stored over 4Å molecular sieves. C_6D_6 and toluene-d_8 were purchased from Goss Scientific Instruments Ltd. and the dried over molten potassium then distilled under argon. [CH{C(Me)NAr}$_2$}Mg^{n}Bu], [CH{C(Me)NAr}$_2$}MgH]$_2$ (Ar = 2,6-^{i}PrC$_6$H$_3$) and were synthesized according to literature procedures.[v]

Synthesis of compound 8

12.4 mg of P_4 (0.1 mmol) added to 0.5 mL of a toluene-d$_8$ solution of 100 mg (^{Dipp}BDI)Mg^{n}Bu (0.2 mmol). As P_4 dissolved the solution turned yellow. NMR analysis after 2 hours at rt showed full and clean conversion to [(^{Dipp}BDI)$_2$Mg$_2$P$_4$Bu$_2$]. The compound crystallized at –36 ^{o}C in the glovebox after addition of a few drops of n-hexanes as colourless crystals (85 mg, 0.15 mmol, 76%). ^{1}H NMR (toluene-d$_8$, 400 MHz, 298K): 6.97-7.08 (m, 6H, Ar-*H*), 4.87 (s, 1H, *β*-C*H*), 3.32, 3.27 (two sept, 2H each, ^{i}Pr-C*H*, ^3J = 6.8 Hz), 1.65 (s, 6H, *α*-C*H$_3$*), 1.28, 1.17, 1.15, 1.13, 1.11 (five d, 1H:1H:2H:1H:1H, ^{i}Pr-C*H$_3$*, ^3J = 6.8 Hz), 1.07-1.31 (m, 12H, ^{n}Bu-C*H$_2$*), 0.88, 0.84 (two t, 3H each, ^{n}Bu-C*H$_3$*, ^3J = 6.8 Hz). ^{13}C{^{1}H} NMR (toluene-d$_8$, 96 MHz, 298K): 169.4 (*C*=N), 146.1 (*i*-Ar-*C*), 142.8, 142.7 (*o*-Ar-*C*), 125.9 (*p*-Ar-*C*), 124.3, 124.1 (*m*-Ar-*C*), 95.5 (*β*-*C*H), 37.8 (broad P*C*H$_2$), 31.4 (broad, PCH$_2$*C*H$_2$), 29.5, 29.1 (^{i}Pr-*C*H), 25.3, 25.0, 24.9, 24.7 (^{i}Pr-*C*H$_3$), 23.8 (*C*H$_2$CH$_3$), 14.5 (^{n}Bu-*C*H$_3$). ^{31}P NMR (toluene-d$_8$, 162 MHz, 298K): –25.6 (d, $^1J_{P-P}$ = 99.6 Hz), –195.5 (d, $^1J_{P-P}$ = 99.6 Hz). Elemental

analysis for [$C_{66}H_{100}Mg_2N_4P_4$] (M_w = 561.0): calc. %C 70.65, %H 8.98, %N 4.99; found %C 70.48, %H 8.83, %N 4.99.

Figure S1: ^{31}P NMR spectrum of compound **8**

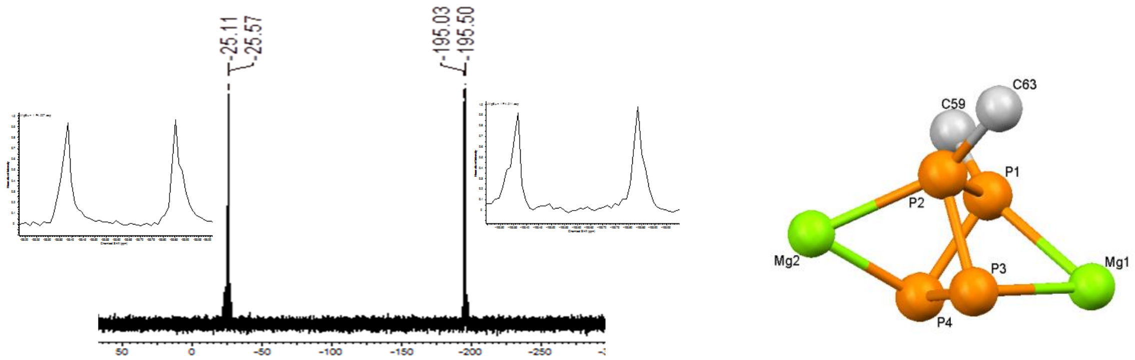

Synthesis of compound 9

24.8 mg of P_4 (0.2 mmol) were loaded into a Youngs tap NMR tube containing 0.5 mL of a C_6D_6 solution of 100 mg (^{Dipp}BDI)Mg*n*Bu (0.2 mmol). The solution slowly turned first yellow then orange as P_4 dissolved. Quantitative conversion to [(^{Dipp}BDI)$_2$Mg$_2$P$_8$Bu$_2$] was achieved after leaving the reaction for 7 days at rt or heating at 60 ^{o}C for 2 days. The compound crystallized upon concentration *in vacuo* as colourless crystals (91 mg, 146 μmol, 73%). Isolated samples of [(^{Dipp}BDI)$_2$Mg$_2$P$_8$Bu$_2$] displayed remarkable thermal stability in toluene-d$_8$ solution, without any sign of decomposition after 2 days at 80 ^{o}C. ^{1}H NMR (C_6D_6, 400 MHz, 298K): 4.90, 4.86 (two s, 1H each, *β*-C*H*), 3.40, 3.34, 3.33, 3.17 (four sept, 2H each, ^{i}Pr-C*H*, ^3J = 6.8 Hz), 1.68, 1.66 (two s, 6H each, *α*-C*H*$_3$), 1.51, 1.44 (two d, 6H each, ^{i}Pr-C*H*$_3$, ^3J = 6.8 Hz), 1.23-1.50 (m, 12H, ^{n}Bu-C*H*$_2$), 1.15, 1.11 (series of overlapping doublets, 36H, ^{i}Pr-C*H*$_3$), 0.88, 0.83 (two t, 3H each, ^{n}Bu-C*H*$_3$, ^3J = 6.8 Hz). ^{13}C{^{1}H} NMR (toluene-d$_8$, 96 MHz, 298K): 170.4 (*C*=N), 146.0 (*i*-Ar-*C*), 143.2, 142.9 (*o*-Ar-*C*), 126.3, 126.2 (*p*-Ar-*C*), 124.9, 124.8, 124.7, 124.3 (*m*-Ar-*C*), 96.0, 95.6 (*β*-*C*H), 36.6, 36.3 (broad P*C*H$_2$), 32.0, 30.9 (broad, PCH$_2$*C*H$_2$), 29.9, 29.6, 29.4, 29.3 (^{i}Pr-*C*H), 26.1, 25.5, 25.4, 25.3 (^{i}Pr-*C*H$_3$), 23.6 (*C*H$_2$CH$_3$), 14.8, 14.5 (^{n}Bu-*C*H$_3$). ^{31}P NMR (C_6D_6, 162 MHz, 298K): 114.0 (ddddd, *P3*, $^1J_{P3-P4}$ = 458 Hz, $^1J_{P3-P2}$ = 385 Hz, $^1J_{P3-P7}$ = 338 Hz, $^2J_{P3-P5}$ = 27 Hz, $^2J_{P3-P1}$ = 8 Hz), 9.1 (dddd, *P1*, $^1J_{P1-P2}$ = 297 Hz, $^1J_{P1-P5}$ = 268 Hz, $^2J_{P1-P6}$ = 86 Hz, $^2J_{P1-P3}$ = 8 Hz), –19.1 (dttm, *P5*, $^1J_{P5-P4}$ = 357 Hz, $^1J_{P5-P1}$ = 268 Hz, $^2J_{P5-P3}$ = 27 Hz), –103.8 (ddm, *P2*, $^1J_{P2-P3}$ = 385 Hz, $^1J_{P2-P1}$ = 297 Hz), –142.9 (dtm, *P7*, $^1J_{P7-P8}$ = 338 Hz, $^1J_{P7-P6}$ = 184 Hz), –158.4 (tddm, *P6*, $^1J_{P6-P8}$ = 268 Hz, $^1J_{P6-}$

$_{P7}$ = 184 Hz, $^{2}J_{P6\text{-}P1}$ = 86 Hz), –217.2 (dd, *P8*, $^{1}J_{P8\text{-}P7}$ = 338 Hz, $^{1}J_{P8\text{-}P6}$ = 268 Hz), –278.3 (ddm, *P4*, $^{1}J_{P4\text{-}P3}$ = 458 Hz, $^{1}J_{P4\text{-}P5}$ = 357 Hz). Elemental analysis for [$C_{66}H_{100}Mg_2N_4P_8$] (M_w = 623.0): calc. %C 63.62, %H 8.08, %N 4.50; found %C 63.48, %H 8.21, %N 4.57.

Figure S2: ^{31}P NMR spectrum of compound **9** with expansions of the signals (clockwise from bottom left) signals at (a) 114.0, (b) 9.1, (c) –19.1, (d) –103.8, (e) –142.9, (f) –158.4, (g) –217.2, (h) –278.3

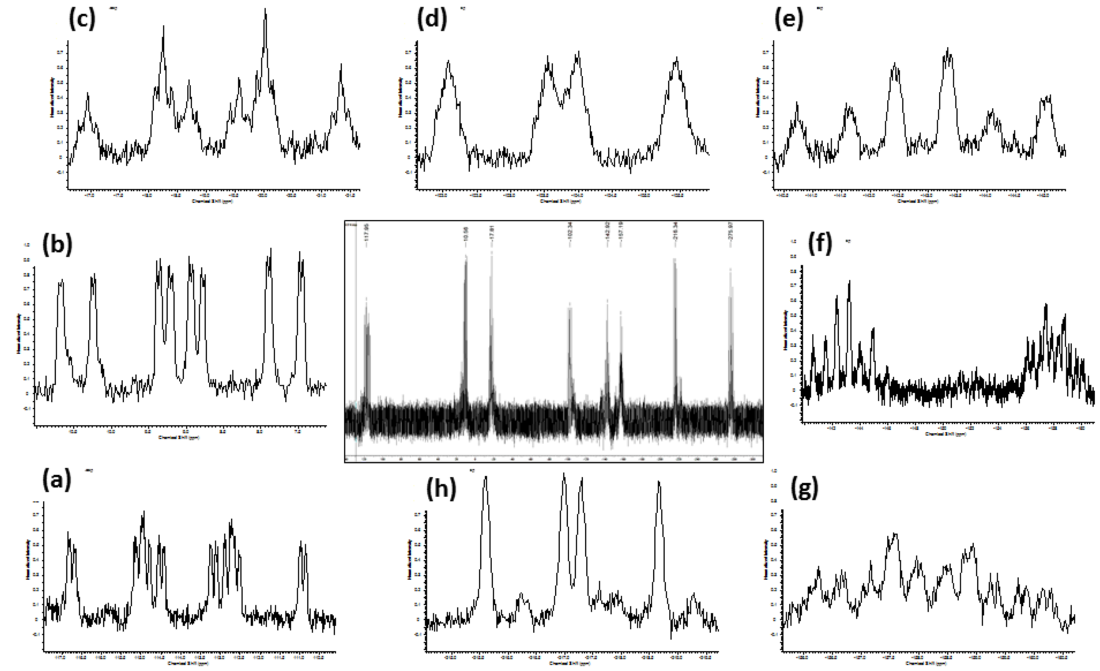

Figure S3: ^{31}P-^{31}P correlation NMR spectrum of compound **9** provided by an *in situ* reaction of compound **8** and P_4 in C_6D_6 solution.

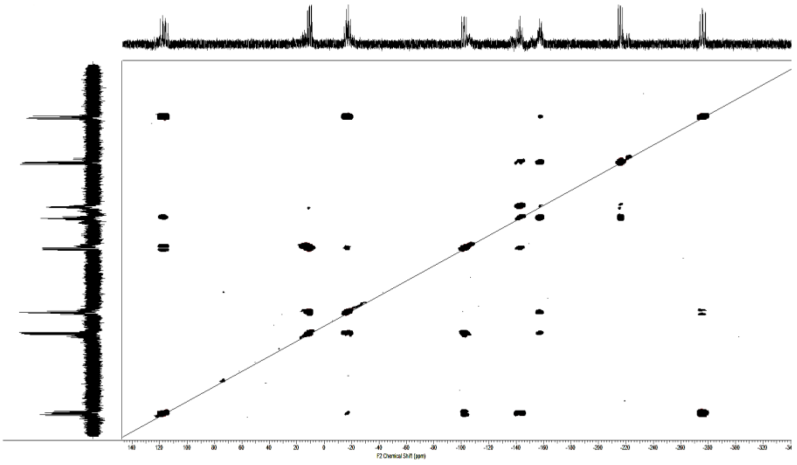

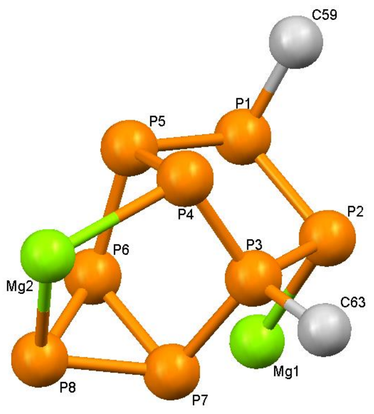

Synthesis of compound 10

Compound **10** was synthesized through the addition of 25 mg of P_4 (0.2 mmol) loaded into a Youngs tap NMR tube containing 0.5 mL of a C_6D_6 solution of 95 mg $[(^{Dipp}BDI)MgH]_2$ (0.2 mmol). The solution slowly turned first yellow then orange as P_4 dissolved and resultant analysis ^{1}H and ^{31}P NMR spectroscopy indicated the formation of a mixture of primarily a species similar to compound **8** and compound **10**. Compound **10** could be isolated and identified as $[(^{Dipp}BDI)MgPH_2]_3$ by an X-ray diffraction analysis as colourless crystals by fractional crystallization from the reaction solution. Attempts to obtain a meaningful elemental analysis and characterization by NMR spectroscopy were frustrated by the decomposition of compound **10** with the formation of PH_3.

10: ^{31}P NMR (C_6D_6, 162 MHz, 298K): δ –284.8 ppm ($^{1}J_{P\text{-}H}$ = 45 Hz)

Figure S4: ^{1}H and $^{31}P\{^{1}H\}$ NMR spectra from the in situ reaction of $[(^{Dipp}BDI)MgH]_2$ and P_4

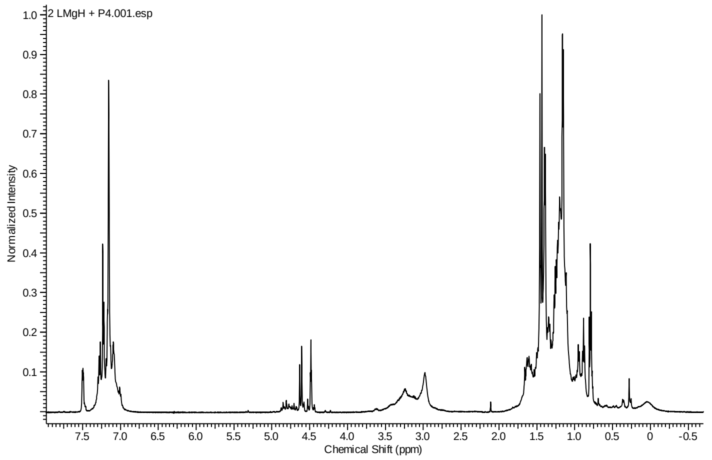

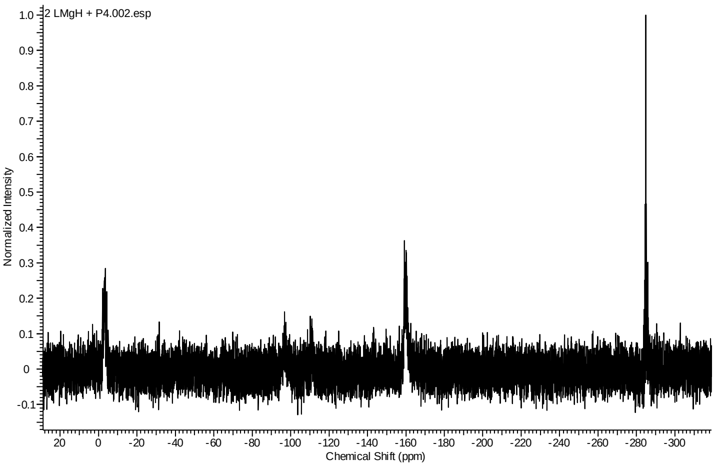

Figure S5: $^{31}P\{^{1}H\}$ and ^{31}P NMR spectra of isolated crystals of compound **3** after several hours illustrating its solution redistribution to PH_3.

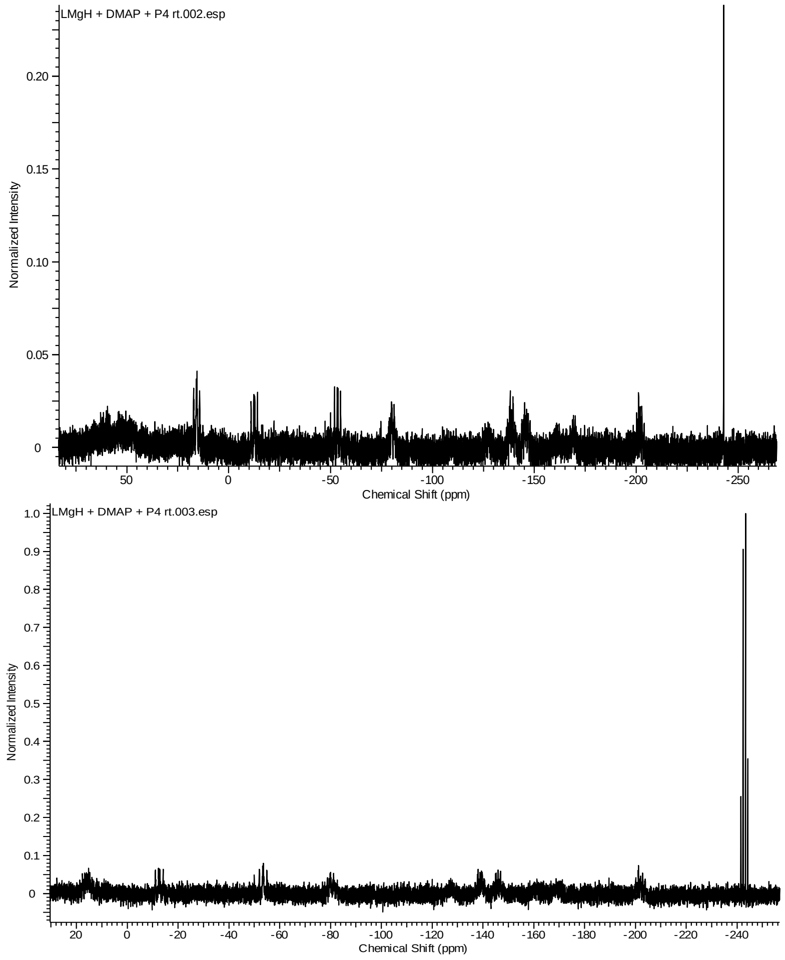

Synthesis of compound 11

44.6 mg of P_4 (0.36 mmol) were loaded into a Youngs tap NMR tube containing 0.5 mL of a toluene-d_8 solution of 100 mg $[(^{Mes}BDI)Mg^{n}Bu]$ (0.24 mmol). The solution slowly turned deep orange as P_4 dissolved. After 5 days at rt (or 2 days at 60 ^{o}C) ^{31}P NMR data indicated full conversion to a complex mixture of at least 3 new phosphorus-containing compounds. After removal of the solvent the mixture was washed with n-hexanes, leaving a yellow solid and an orange filtrate. Crystals of $[(^{Mes}BDI)_3Mg_3P_7]$ were obtained from the filtrate after 1 day at rt (ca. 5 mg, 4 μmol, 5% based on Mg). ^{1}H NMR (C_6D_6, 500 MHz, 298K): 7.00 (s, 4H, *m*-Mes-*H*), 4.69 (s, 1H, *β*-C*H*), 2.38 (s, 6H, *p*-C*H*$_3$), 2.15 (s, 12H, *o*-C*H*$_3$), 1.51 (s, 6H, *α*-C*H*$_3$). $^{13}C\{^{1}H\}$ NMR (C_6D_6, 126 MHz, 298K): 168.2 (N=*C*), 147.2 (*i*-Mes-*C*), 133.3 (*p*-Mes-*C*), 132.3 (*o*-Mes-*C*), 130.5 (*m*-Mes-*C*), 94.7 (*β*-*C*H), 24.0 (*α*-*C*H$_3$), 21.8 (*p*-*C*H$_3$), 20.4 (*o*-*C*H$_3$). ^{31}P NMR (C_6D_6, 202 MHz, 298K): –95.3 (very broad m, 1P, *P4*), –100.3

(broad m, 3H, *P5/6/7*), –158.3 (broad m, *P1/2/3*). A meaningful elemental analysis could not be obtained for this compound.

X-ray crystallography

The X-ray data for compound **9** indicated that sample was non-merohedrally twinned. The relationship between the orientation matrices of the 2 approximately equal twin components is given by a rotation if 180° around -0.71 0.00 0.71 (reciprocal space vector) or -0.86 -0.00 0.52 (direct space direction). The overlap between the twinned diffraction patterns is in the region of 21%. Crystal solution proceeded without incident using data from one of the twinned components. Refinement is based on the twinned data set from both species, and affords a credible result. The residuals are slightly higher than desirable, and this largely reflects a small amount of disorder in the phosphorus region, which has a knock on effect on the associated butyl groups based on C59 and C63. Strenuous efforts were made to model this disorder but, as it is in the region of 10-15% it proved impossible and hence was abandoned. Bonded C-C distances in addition to some non-bonded C1...C3 distances were restrained within the pendant butyl groups, in the final least squares cycles, to assist convergence. Additionally, data were truncated due to a fall-off of intensity at the highest Bragg angles.

i A. Altomare, M. C. Burla, M. Camalli, G. L. Cascarano, C. Giacovazzo, A. Guagliardi, A. G. G. Moliterni, G. Polidori and R. Spagna, *Journal of Applied Crystallography*, **1999**, *32*, 115-119.

ii Z. Otwinowski and W. Minor, *DENZO-SMN Manual*, University of Texas Southwestern Medical Center, Dallas, USA, **1996**.

iii G. M. Sheldrick, *SHELXL97-2*, *Program for Crystal Structure Refinement*, Universität Göttingen, Göttingen, Germany, **1998**.

iv C. Barnes, *Journal of Applied Crystallography*, **1997**, *30*, 568.

v (a) V. C. Gibson, J. A. Segal, A. J. P. White, D. J. Williams,*J. A. Chem. Soc.* **2000**, *122*, 7120-7121; (b) S. J. Bonyhady, C. Jones, S. Nembenna, A. Stasch, A. J. Edwards, G. J. McIntyre, *Chem. Eur. J.* **2010**, *16*, 938-955.
